# Individual- and neighborhood-level characteristics of lung cancer screening participants undergoing telemedicine shared decision making

**DOI:** 10.1186/s12913-023-10185-4

**Published:** 2023-10-30

**Authors:** Christine S. Shusted, Hee-Soon Juon, Brooke Ruane, Brian Till, Charnita Zeigler-Johnson, Russell K. McIntire, Tyler Grenda, Olugbenga Okusanya, Nathaniel R. Evans, Gregory C. Kane, Julie A. Barta

**Affiliations:** 1 Division of Pulmonary and Critical Care Medicine, The Jane and Leonard Korman Respiratory Institute at Thomas Jefferson University, 834 Walnut Street, Suite 650, Philadelphia, PA 19107 USA; 2 Department of Medical Oncology, Division of Population Science, Thomas Jefferson University, Philadelphia, PA 19107 USA; 3 Division of Thoracic Surgery, The Jane and Leonard Korman Respiratory Institute at Thomas Jefferson University, Philadelphia, PA 19107 USA; 4 Jefferson College of Population Health, Thomas Jefferson University, Philadelphia, PA 19107 USA

**Keywords:** Lung cancer screening, Telemedicine, Health disparities, Lung cancer, Screening adherence

## Abstract

**Background:**

Although lung cancer screening (LCS) for high-risk individuals reduces lung cancer mortality in clinical trial settings, many questions remain about how to implement high-quality LCS in real-world programs. With the increasing use of telemedicine in healthcare, studies examining this approach in the context of LCS are urgently needed. We aimed to identify sociodemographic and other factors associated with screening completion among individuals undergoing telemedicine Shared Decision Making (SDM) for LCS*.*

**Methods:**

This retrospective study examined patients who completed Shared Decision Making (SDM) via telemedicine between May 4, 2020 – March 18, 2021 in a centralized LCS program. Individuals were categorized into Complete Screening vs. Incomplete Screening subgroups based on the status of subsequent LDCT completion. A multi-level, multivariate model was constructed to identify factors associated with incomplete screening.

**Results:**

Among individuals undergoing telemedicine SDM during the study period, 20.6% did not complete a LDCT scan. Bivariate analysis demonstrated that Black/African-American race, Medicaid insurance status, and new patient type were associated with greater odds of incomplete screening. On multi-level, multivariate analysis, individuals who were new patients undergoing baseline LDCT or resided in a census tract with a high level of socioeconomic deprivation had significantly higher odds of incomplete screening. Individuals with a greater level of education experienced lower odds of incomplete screening.

**Conclusions:**

Among high-risk individuals undergoing telemedicine SDM for LCS, predictors of incomplete screening included low education, high neighborhood-level deprivation, and new patient type. Future research should focus on testing implementation strategies to improve LDCT completion rates while leveraging telemedicine for high-quality LCS.

**Supplementary Information:**

The online version contains supplementary material available at 10.1186/s12913-023-10185-4.

## Introduction

In the years since the National Lung Screening Trial, and subsequent revisions to the United States Preventive Services Task Force (USPSTF) lung cancer screening (LCS) guidelines, LCS uptake has slowly increased, with some states now reporting up to 15% of potentially eligible, high-risk individuals undergoing annual low-dose CT (LDCT) scan [1–4]. Initial challenges in LCS implementation included the development of multidisciplinary programs, achieving buy-in from stakeholders, and reliable tracking of LCS results, among other barriers [5]. On an annual basis, the screening process includes confirmation of eligibility, shared decision-making (SDM), LDCT scan, and results review followed by appropriate evaluation of screen-detected lung nodules and incidental findings [6]. As LCS programs have grown, new challenges have emerged. These include improving screening adherence and increasing equitable LCS uptake and completion of the screening process, especially among vulnerable populations [6–9]. Moreover, the overall population eligible for screening has increased substantially with the United States Preventive Services Task Force (USPSTF) 2021 recommendations, specifically among women and racial/ethnic minority groups [10, 11].

The COVID-19 pandemic introduced additional challenges for healthcare providers and has led to decreased rates of screen-detected cancer diagnoses and disruptions in care for cancer screening programs [12–14]. In the context of lung cancer, an expert panel from the American College of Chest Physicians recommended deferment of LCS enrollment and surveillance imaging for incidental lung nodules during the pandemic [15]. Data on the impact of these modifications remains limited. Although some programs have successfully returned to pre-pandemic levels of LCS, others report that rates of Lung-RADS Category 4 results have increased, and no-show rates remain higher than pre-pandemic levels [16–18].

Upon resuming routine LCS, many healthcare systems pivoted to telemedicine for shared decision-making (SDM) to minimize COVID-19 transmission risk for patients and providers. This pivot was noted by major payers including the Centers for Medicare and Medicaid Services (CMS), and several private insurers, which lead to relaxing telemedicine restrictions in place pre-COVID-19 and increasing reimbursement levels [19].

This change in practice may lead to a cascade of alterations in LCS implementation and has the potential to exacerbate disparities in screening [20]. In the setting of recent dramatic increases in telemedicine usage, racial and socioeconomic disparities in utilization have been noted in multiple contexts, including COVID-related care, primary care, and subspecialty care [21–24]. For centralized LCS programs, data remain limited [18]. For example, it is not known if implementation of LCS via telemedicine introduces barriers for vulnerable populations who already experience disparities in cancer screening [10].

The objective of this retrospective study was to characterize individuals undergoing LCS with SDM via telemedicine as well as to identify potential differences in those who did and did not complete a LDCT scan after SDM. We hypothesized that sociodemographic characteristics of individuals who completed the entire LCS process would be significantly different from those of individuals who underwent SDM, but did not obtain a LDCT scan.

## Methods and materials

### Lung cancer screening program and study population

All patients in this study were enrolled in the Jefferson Lung Cancer Screening Program; a centralized LCS program at a major urban academic medical center. and were identified through the Program’s LCS Registry version 2021Q2. The Jefferson LCS Program has been screening USPSTF 2013-, CMS-, and/or NCCN-eligible (National Comprehensive Cancer Network) patients through a centralized nursing-driven model since 2015. Dedicated program staffing is comprised of: 1). A Coordinator who manages referrals, schedules patients and obtains insurance authorization; 2). Two Nurse Navigators who are clinical nurse specialists and assist with SDM, perform tobacco treatment counseling, and review screening results with patients and primary care providers; and 3) A Nurse Practitioner who carries out SDM, organizes diagnostic evaluation for screening patients who require additional testing and procedures, and manages the day-to-day activities of the LCS program. Patients are electronically referred by primary care providers, pulmonologists, or other specialists, but can also self-refer to the LCS Program, and screening eligibility is confirmed by the Coordinator. SDM is carried out by the Nurse Navigator and Nurse Practitioner as described below. All positive screening LDCTs are reviewed by a multi-disciplinary team on a weekly basis, and detailed recommendations on management of screen-detected nodules and workup of incidental findings are communicated to both the patient and the referring primary care provider by the Nurse Navigators and Nurse Practitioner. A standardized intake form is used to collect demographic and clinical data at the time of entry into the LCS Program and is updated prospectively with screening results and subsequent workup. Accuracy of entered data is confirmed by random chart review, and clinical outcomes are updated, both on a quarterly basis. This study protocol – was reviewed and approved by the Thomas Jefferson University Institutional Review Board (IRB) with a waiver of informed consent, given the minimal risk nature of the study (IRB Control#, 17D.150). This retrospective analysis was carried out in accordance with all methodological guidelines and regulations of the Thomas Jefferson University IRB, as well as the Strengthening the Reporting of Observational Studies in Epidemiology (STROBE) reporting guideline.

Our LCS Program paused screening of new patients during the period March 18, 2020 – May 4, 2020, due to the first surge of the COVID-19 pandemic. For this study, all individuals who completed SDM and agreed to LCS with LDCT following LCS Program reopening were identified as the Post-Telehealth SDM cohort (between May 4, 2020 and March 18, 2021). An additional cohort of patients who completed SDM and LCS in the year prior to the study period (May 4, 2019 and March 18, 2020) were identified as the Pre-Telehealth SDM cohort. Individuals residing outside of Philadelphia were excluded from the study to allow for geospatial analyses of Philadelphia neighborhoods. Patients’ sociodemographic information, medical history, and screening-related outcomes were obtained from the LCS Program Registry database.

### Shared decision-making

The LCS Program offers SDM that is tailored to patients’ health literacy and general understanding of cancer screening. The Nurse Navigator, supervised by the Nurse Practitioner, reviews basic principles of lung cancer screening with LDCT and discusses potential harms and benefits. Patients are given ample opportunity to raise questions and discuss concerns. Tobacco treatment counseling and pharmacotherapy prescriptions are also provided for individuals who currently smoke. Following the SDM visit, patients elect to proceed or decline screening. For patients who choose to undergo LCS, the LDCT is coordinated by the LCS Program and screening results are reviewed by telephone with patients and by electronic communication primary care providers.

Prior to the COVID-19 pandemic, appointments for same-day, in-person SDM and LDCT were scheduled by the LCS Program Coordinator. Following the Program reopening, all SDM was conducted via telemedicine, and LDCT appointments were scheduled immediately afterward by the Coordinator. Decision aids were offered electronically or by mail, and at the end of the SDM appointment individuals were scheduled for an LDCT scan (with most appointments within 7–14 days of SDM). Individuals who missed their LDCT scan received a follow-up telephone call and a reminder letter to reschedule the LDCT appointment.

### Outcomes

Individuals who underwent SDM via telemedicine were examined for the primary outcome, LDCT completion, and the study cohort was divided into two subgroups for comparison. The Complete Screening Subgroup included individuals who underwent SDM during the study period and subsequently completed LCS with LDCT. The Incomplete Screening Subgroup included individuals who underwent SDM during the same period, but did not follow up with a LDCT scan. Incomplete screening was defined as not undergoing screening LDCT within 1 month of completing SDM through our LCS Program. Factors associated with incomplete screening were identified using logistic regression analyses.

Screening adherence was determined using modified National Lung Cancer Roundtable (NLCRT) definitions [9, 25]. For individuals due for annual screening (Lung-RADS 1 or 2), a return date of 12 months rather than 18 months was used due to the timeframe of the study period. For individuals with a positive Lung-RADS score, NLCRT quality metric definitions were followed, meaning Lung-RADS Category 3 had a return date of 6 – 8 months was used, and Lung-RADS 4A had a return date of 3 – 4.5 months [7].

### Neighborhood deprivation index, geocoding, and mapping

Neighborhood Deprivation Index (NDI) values for Philadelphia census tracts were obtained from a publicly available dataset via the National Cancer Institute’s GIS Portal for cancer research [26]. The NDI includes 13 measures across various facets of socioeconomic status based on 5-year American Community Survey data. Areas of focus include wealth and income, education, occupation, and housing conditions [26–28]. NDI scores are provided in a continuous form with values ranging from -2.5 – 1.9, with higher values indicating greater neighborhood deprivation, as well as in quintiles (Least Deprivation – Most Deprivation) [28].

For all individuals, home address was collected from the LCS Registry and geocoded by census tract utilizing ArcGIS Pro [29]. NDI values were joined with patient count at the census level to visualize differences between the Complete Screening and Incomplete Screening subgroups. Distance from each screening participant’s home address to our LCS site was calculated for each patient. Distance analyses were performed with ArcGIS Pro and Google Maps [29, 30]. Distance was measured via street network in miles, the number of minutes by car, and the number of minutes by mass transit. Consistent with accepted methods in the literature, transit times (vehicle and mass transit) were calculated at a standardized time and day to ensure equal conditions across patients [31].

### Statistical analysis

We obtained descriptives, frequencies and cross-tabulations to summarize descriptive data in tables. Bivariate analyses including independent t-tests and chi-square tests were performed to examine characteristics of the Complete Screening and Incomplete Screening subgroups using a p ≤ 0.05 significance threshold. Bivariate logistic regression analysis was conducted for individual characteristics (e.g., age, gender, race, education, smoking status, type of visit). With a multilevel data structure of neighborhood-level of NDI (e.g., patients nested within census tracts), multivariate and multilevel analysis was conducted to account for clustering effect. SPSS version 26 was used, with STATA version 17 used for predictive modeling [32, 33].

## Results

### Baseline characteristics

A total of 754 unique individuals underwent telemedicine SDM for LCS through the Jefferson Lung Cancer Screening Program during the study period (Table 1). The majority of individuals were female (*n* = 429, 56.9%) and self-reported their race as White (*n* = 416, 55.2%). Over half of the cohort reported current tobacco use (*n* = 422, 56.0%), and had a mean smoking intensity of 51.96 ± 23.50 pack-years. A high school diploma or GED equivalent was the most commonly reported level of educational attainment (*n* = 350, 46.4%) and a plurality of patients reported being privately insured (*n* = 287, 38.1%).

**Table 1:** Baseline characteristics of individuals who underwent SDM with incomplete screening and complete screening subgroups

	**Patients Who Underwent Shared Decision Making For Lung Cancer Screening (*n* = 754)**		**Incomplete Screening Subgroup (*n* = 155)**		**Complete Screening Subgroup (*n* = 599)**		***p*-value**^**a**^
**Age, mean (SD)**	63.93	(5.73)	63.47	(5.75)	64.05	(5.72)	0.265
**Gender, n (%)**							0.136
Female	429	(56.9%)	80	(51.6%)	349	(58.3%)	
Male	325	(43.1%)	75	(48.4%)	250	(41.7%)	
**Ethnicity, n (%)**							0.790
Hispanic/Latinx	27	(3.6%)	5	(3.2%)	22	(3.7%)	
**Race, n (%)**							< 0.001
Black/African-American	307	(40.7%)	82	(52.9%)	225	(37.6%)	
White	416	(55.2%)	59	(38.1%)	357	(59.6%)	
Other^b^	31	(4.1%)	14	(9.0%)	17	(2.8%)	
**Smoking status, n (%)**							0.683
Current	422	(56.0%)	89	(57.4%)	333	(55.6%)	
Former	332	(44.0%)	66	(42.6%)	266	(44.4%)	
**Pack-years, mean (SD)**	51.96	(23.50)	52.38	(23.47)	50.32	(23.63)	0.219
**Personal history of cancer, n (%)**	133	(17.6%)	16	(10.3%)	116	(19.4%)	0.008
**Family history of lung cancer, n (%)**	219	(29.0%)	27	(17.4%)	192	(32.1%)	0.047
**COPD, n (%)**	408	(54.1%)	61	(39.4%)	347	(57.9%)	< 0.001
**BMI, mean (SD)**	28.61	(7.70)	27.97	(8.05)	28.77	(7.61)	0.255
**Education, n (%)**							0.023
< HS Diploma	80	(10.6%)	21	(13.5%)	59	(9.8%)	
HS Diploma/GED	350	(46.4%)	67	(43.2%)	283	(47.2%)	
> HS Diploma	287	(38.1%)	53	(34.2%)	234	(39.1%)	
Unknown	37	(4.9%)	14	(9.0%)	23	(3.8%)	
**Insurance Status, n (%)**							0.042
Medicare	278	(36.9%)	44	(28.4%)	234	(39.1%)	
Medicaid/Dual Eligible	189	(25.1%)	47	(30.3%)	142	(23.7%)	
Private/Other^c^	287	(38.1%)	62	(40.0%)	206	(34.4%)	
**PLCOm2012 Risk, mean (SD)**	6.34	(5.64)	6.46%	(5.85)	5.71%	(4.37)	0.929
**Patient Type, n (%)**							
New Patient	360	(47.7%)	90	(58.1%)	270	(45.1%)	0.001
Return Patient	394	(52.3%)	65	(41.9%)	329	(54.9%)	

^a^*P*-value indicates differences between Incomplete Screening and Complete Screening cohorts

^b^Other race includes individuals who reported their race as Asian, Alaskan Native/American Indian, Native Hawaiian/Pacific Islander, or More than One Race

^c^Private/Other insurance includes Private Insurance, State Marketplace, and Workers Compensation Plans

### Sociodemographic characteristics of the complete screening and incomplete screening subgroups

Among the 754 individuals completing telemedicine SDM, 599 patients (79.4%) underwent LDCT (comprising the Complete Screening Subgroup) and 155 patients (20.6%) did not undergo LDCT scan (comprising the Incomplete Screening Subgroup) (Table 2). The Incomplete Screening Subgroup had a significantly greater proportion of Black/African American individuals compared to the Complete Screening Subgroup (52.9% vs. 37.6%, p < 0.001). The Complete Screening Subgroup reported a significantly higher rate of personal history of cancer, family history of lung cancer, and COPD compared with the Incomplete Screening Subgroup. There was a statistically significant difference in educational attainment between the two subgroups, with a greater frequency of individuals with less than a high school (HS) diploma or GED equivalent in the Incomplete Screening Subgroup compared with the Complete Screening Subgroup (13.5% vs. 9.8%), and a lesser proportion of individuals with at least a HS diploma/GED (77.4% vs. 86.3%, respectively). Patients in the Incomplete Screening Subgroup reported having Medicaid significantly more than those in the Complete Screening Subgroup. The Incomplete Screening Subgroup was made up of a significantly greater proportion of new patients undergoing first-time SDM compared with the Complete Screening Subgroup (58.1% vs. 45.1%, *p* = 0.001). There was no significant difference in distance to screening site between the two subgroups (Supplemental Table 1).

**Table 2:** Sociodemographic factors associated with incomplete screening among individuals undergoing lung cancer screening

	**Bivariate Logistic Regression** **Unadjusted OR (95% CI)**		**Multi-level Multivariate Analysis** **Adjusted OR (95% CI)**	
**Age**	1.005	(0.975–1.037)	0.977	(0.964–0.989)**
**Gender**				
Female	0.764	(0.536–1.089)	0.868	(0.597–1.263)
Male	1.000		1.000	
**Race**^**a**^				
White	1.000		1.000	
Black/African-American	2.162	(1.489–3.138)**	1.177	(0.788–1.758)
**Smoking Status**				
Current	1.077	(0.754–1.539)	0.978	(0.671–1.425)
Former	1.000		1.000	
**Education**^**a**^				
< HS Diploma	1.000		1.000	
HS Diploma/GED	0.653	(0.371–1.149)	0.573	(0.330–0.994)*
> HS Diploma	0.651	(0.365–1.162)	0.587	(0.327–1.051) +
**Insurance Status**				
Medicare	1.000		1.000	
Medicaid/Dual Eligibility	1.713	(1.083–2.712)*	1.257	(0.784–2.014)
Private/Other^b^	1.456	(0.953–2.226) +	1.239	(0.804–1.907)
**Visit Type**				
New patient	1.687	(1.180–2.412)**	1.505	(1.039–2.179)*
Return patient	1.000		1.000	
**Neighborhood Deprivation**				
NDI	1.224	(0.993–1.509) +	1.784	(1.320–2.412)*

^+^*p* ≤ 0.10; *p ≤ 0.05; **p ≤ 0.01

^a^Individuals with Other race (including Asian, Alaskan Native / American Indian, Native Hawaiian / Pacific Islander, or More than One Race) and/or Unknown education were excluded from this analysis (*n* = 59)

^b^Private / Other insurance includes Private plans, State Marketplace, Self-Pay, Uninsured, and Workers Compensation Plans

### Neighborhood deprivation index among the complete screening and incomplete screening subgroups

The Incomplete Screening Subgroup had a significantly higher mean NDI (greater socioeconomic deprivation) compared with the Complete Screening Subgroup (*p* < 0.001) (Supplemental Table 1). Upon comparison of NDI, quintiles 54.2% of the Incomplete Screening Subgroup resided in census tracts with above average or greater deprivation, while 45.4% of the Complete Screening Subgroup resided in census tracts with above average or greater deprivation.

**Fig. 1:**
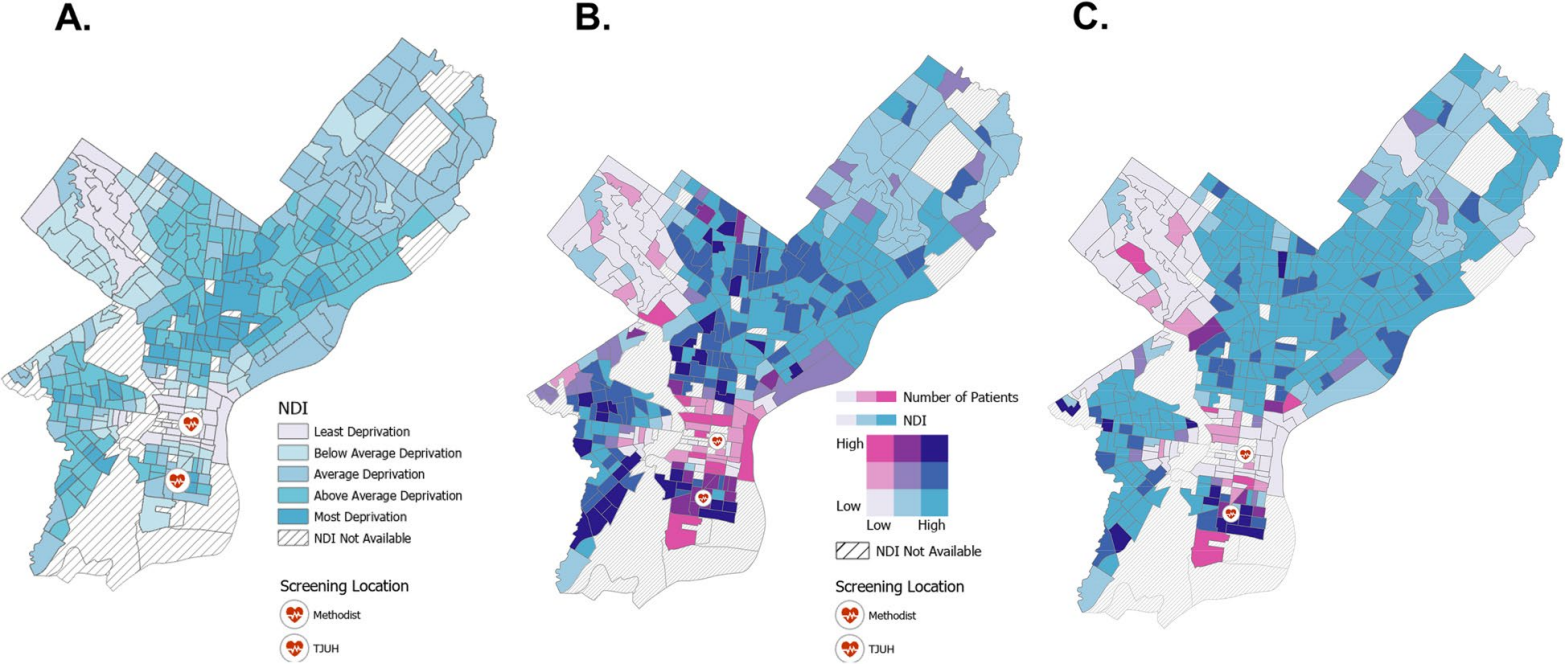
Neighborhood Deprivation Index (NDI) by Philadelphia Census Tract. **A** NDI quartiles **B**). Bivariate choropleth map of NDI and patient count among the Complete Screening Subgroup. **C** Bivariate choropleth map of NDI and patient count among the Incomplete Screening Subgroup. Maps were generated using ArcGIS Pro, 2.5 Ed. Redlands, CA; ESRI, 2020

Pre-defined NDI quartiles are displayed by Philadelphia census tract in Fig. 1A. Utilizing bivariate choropleth mapping, Fig. 1B and C demonstrate LCS program patients and NDI by census tract. Tracts in dark pink represent areas with a relative high number of screening patients and low NDI, while tracts in turquoise represent areas with few patients but a high NDI. The turquoise tracts show consistent disparity across a wide array of socioeconomic measures and are located primarily in North and West Philadelphia. Compared with the Complete Screening Subgroup, the Incomplete Screening Subgroup includes a greater proportion of individuals from Philadelphia census tracts with a high level of neighborhood deprivation (Fig. 1 and Supplemental Table 1).

### Factors associated with incomplete screening among individuals undergoing telemedicine shared decision making

Sociodemographic characteristics of the individuals who received SDM via telemedicine during the study period were analyzed to identify factors associated with incomplete screening (SDM completion without subsequent LDCT). Black/African-American race, new patient type, and Medicaid insurance status were significantly associated with greater odds of incomplete screening (Table 2). Upon adjustment for covariates and for clustering effects in a multivariate, multi-level analysis, individuals entering the screening program for the first time had significantly higher odds of incomplete screening (aOR 1.51; 95%CI, 1.04–2.18). Individual education attainment (HS Diploma, aOR 0.57; 95%CI, 0.33–0.99) and Philadelphia census tract NDI (aOR 1.78; 95%CI, 1.32–2.41) were also significantly associated with incomplete screening status (Table 2).

### Comparison of patients pre & post telehealth shared decision making

A total of 833 patients underwent SDM in-person for LCS prior to the pivot to telemedicine SDM, in the ten months following the implementation of telemedicine SDM, 754 patients completed SDM. There were no statistically significant differences between the Pre-Telehealth SDM and Post-Telehealth SDM cohorts with regard to age, gender, ethnicity, race, smoking status, BMI, insurance status, or PLCOm2012 lung cancer risk (Supplemental Table 2). There was a statistically significant difference (*p* < 0.001) in educational attainment between the two groups, with a lower frequency of individuals with *less than* a high school (HS) diploma screened in the Post-Telehealth SDM cohort compared with the Pre-Telehealth SDM cohort (10.6% vs. 16.1%, respectively) and a higher frequency of individuals *with* a HS diploma (46.4% vs. 41.7%, respectively). Among the Post-Telehealth SDM patients, there was a lower frequency of individuals with the highest level of educational attainment (*greater than* a HS diploma), at 38.1% vs. 40.3%, respectively. The Post-Telehealth SDM cohort also had a statistically significantly higher rate of COPD (54.1% vs. 44.7%, *p* < 0.001) and lower smoking intensity compared with the Pre-Telehealth SDM cohort (51.96 vs. 54.42 pack-years, *p* = 0.046). The majority of individuals in the Pre-Telehealth SDM cohort were new patients (73.3%), whereas the majority of patients seen after the transition to telehealth SDM were returning patients (52.3%). A significantly greater proportion of the Post-Telehealth SDM cohort lived in neighborhoods with at least above-average deprivation compared to the Pre-Telehealth SDM cohort (47.3% vs. 41.8%; *p* = 0.030).

## Discussion

This retrospective analysis of individuals undergoing telehealth SDM as part of LCS is a unique perspective on the impact of telemedicine on screening among high-risk individuals. We identified a new potential pitfall of telemedicine SDM implementation with 20% of individuals not returning for SDM, which we described as incomplete screening. Predictors of incomplete screening on multivariate, multi-level analysis included educational attainment, new patient type, and Philadelphia census tract NDI. A GIS analysis of NDI and screening patient count in Philadelphia revealed geographic areas of consistent disparity and low screening.

Our centralized LCS program previously offered same-day in-person SDM and LDCT, and we and others have demonstrated that more than 90% of individuals who undergo in-person SDM will complete LDCT screening [34, 35]. Following our Program’s pivot to telehealth, SDM occurred days to weeks ahead of the LDCT scan, allowing for attrition. Literature suggests that telemedicine ambulatory visits are canceled significantly less frequently than in-person visits, however, that does not account for the additional required in-person aspect of undergoing a CT scan [36]. The implementation of telehealth SDM provides patients with more time to complete their decision on whether or not to be screened and avoid feelings of pressure to undergo LDCT immediately, it also creates an opportunity for patients to forget their LDCT appointment. While this study was not designed to assess intent to screen or to determine reasons for incomplete screening – including whether patients are lost because of the telemedicine approach or due to other reasons entirely – future research should identify these factors to mitigate incomplete screening.

These results demonstrate that some populations undergoing SDM may be at risk for incomplete screening. Directionality of the NDI at the census tract level was consistent with that of individual-level education, with both lower education and greater socioeconomic deprivation being significantly associated with incomplete screening. This is similar to existing literature that has demonstrated that individuals with lower levels of education may report finding telemedicine platforms difficult to navigate [37]. The literature also suggests that as educational attainment increases, so does telehealth uptake; however, we found the opposite when examining the pre and post telehealth SDM cohorts [37, 38]. In contrast, NDI analysis demonstrated that there was an increase in individuals from census tracts with the greatest level of deprivation in the Post-Telehealth SDM cohort. This difference may reflect that the NDI is a more comprehensive measure of socioeconomic status compared with educational attainment alone, or that interactions may exist between individual- and neighborhood-level characteristics.

In addition, new patient status was also significantly associated with incomplete screening. Similar to other programs reporting LCS volumes during the COVID-19 pandemic, we observed a decrease in new patients undergoing baseline LDCT and an increase in returning patients [18]. This may be because returning patients are familiar with the LCS process and have an existing relationship with the program. New patients might have been more apprehensive about undergoing a preventive measure with new providers during a pandemic. In response to this finding, our LCS Program now requires in-person SDM for all new LCS individuals undergoing baseline LDCT.

The main limitation of this study is its retrospective, single-center design amid a global healthcare crisis. The COVID-19 pandemic makes it difficult to differentiate the effects of our LCS Program’s pivot to telemedicine from the overall impact of the pandemic itself. Although this study reports findings from a single LCS program, the patient population reflects the racial diversity of Philadelphia and therefore, the results may be applicable to other centralized programs at urban, academic institutions. Strategies to reduce incomplete screening are essential for high-quality LCS, and additional studies to test our findings in other populations are also needed. Second, although distance to the screening center did not significantly differ between cohorts, there are limitations within the distance analyses. Patients were not surveyed about how they traveled to the screening site and from where. Although almost all patients underwent LCS at our main Radiology site a small minority of patients may have undergone screening at locations closer to their homes or workplaces, and these data were not extracted from the electronic health record. Future studies should survey patients on their transportation methodology ahead of distance calculations.

Our results suggest that socioeconomic and other factors may influence the implementation of telemedicine approaches for LCS. Specifically, telemedicine may be a barrier to high-quality LCS for individuals with low levels of educational attainment or high neighborhood deprivation, who are entering a screening program for the first time. Importantly, our results also underscore that LCS is a multi-step process and that telemedicine SDM, while enhancing access for some high-risk individuals, may also create an additional barrier leading to incomplete screening for others. Providers and programs utilizing telemedicine for LCS must carefully consider how to effectively reach all individuals, but especially members of underserved populations. Underscoring the responsibility of incorporating telemedicine into screening programs properly, is that in-person counseling has been shown to diagnosis a greater proportion of lung cancers compared to counseling performed over a telehealth platform [39]. As telemedicine remains a critical part of healthcare delivery in the US and worldwide, prospective research is urgently needed to determine how telemedicine impacts screening implementation among LCS-eligible populations. Future work should focus on vulnerable individuals to ensure equitable delivery of LCS services and mitigate disparate outcomes in LCS.

## Conclusion

Among high-risk individuals who completed SDM via telehealth, we described a newly identified pitfall, a clinically significant percentage of patients who participated in SDM did not obtain a LDCT scan, thus did not complete the LCS process. Significantly, fewer patients with the lowest levels of educational attainment completed a LDCT, placing an already high-risk population, at an even greater risk. After adjustment for covariates, predictors of incomplete screening via telemedicine included low education, high neighborhood-level deprivation, and new patient type.

### Supplementary Information

**Additional file 1:** **Supplemental Table 1.** Distance to Screening Site and Neighborhood Deprivation Index (NDI) Among Individuals in the Incomplete Screening and Complete Screening Subgroups. **Supplemental Table 2.** Baseline Characteristics Of Individuals Receiving Shared Decision Before and After Telehealth Implementation.

## Data Availability

The datasets used and/or analyzed during the current study are available from the corresponding author on reasonable request.

## Abbreviations

LCS: Lung Cancer Screening
LDCT: Low-Dose CT
USPSTF: United States Preventive Services Task Force
SDM: Shared Decision-Making
NDI: Neighborhood Deprivation Index
