# Supplementary material for: Individual- and neighborhood-level characteristics of lung cancer screening participants undergoing telemedicine shared decision making
Source: BMC Health Serv Res. 2023 Oct 30;23:1179. doi: 10.1186/s12913-023-10185-4 (PMC10614340; doi:10.1186/s12913-023-10185-4)
Supplement: Supplementary file 1 — Additional file 1: Supplemental Table 1. Distance to Screening Site and Neighborhood Deprivation Index (NDI) Among Individuals in the Incomplete Screening and Complete Screening Subgroups. Supplemental Table 2. Baseline Characteristics Of Individuals Receiving Shared Decision Before and After Telehealth Implementation. [file 12913_2023_10185_MOESM1_ESM.docx]

| **Supplemental Table 1. Distance to Screening Site and Neighborhood Deprivation Index (NDI)** | | | | | |
| --- | --- | --- | --- | --- | --- |
|  | **Incomplete Screening Subgroup**  **(n=155)** | | **Complete Screening Subgroup**  **(n=599)** | | **p-value** |
| **Distance to Screening Site** | | | | | |
| **Distance to TJUH**  Street Network in Miles, mean (SD) | 6.95 | (9.26) | 7.71 | (12.37) | 0.477 |
| **Time to TJUH** |  |  |  |  |  |
| By Car, in Minutes, mean (SD) | 22.56 | (11.89) | 23.53 | (16.08) | 0.480 |
| By Mass Transit, in Minutes, mean (SD) | 36.74 | (29.71) | 39.09 | (40.32) | 0.497 |
| **Neighborhood Deprivation Index* (NDI)** | | | | | |
| **NDI, mean (SD)** | 0.426 | (1.13) | 0.131 | (0.758) | < 0.001 |
| **NDI National Quintiles, n (%)** |  |  |  |  | 0.009 |
| Least Deprivation | 16 | (10.3%) | 79 | (13.2%) |  |
| Below Average Deprivation | 12 | (7.7%) | 77 | (12.9%) |  |
| Average Deprivation | 22 | (14.2%) | 116 | (19.4%) |  |
| Above Average Deprivation | 16 | (10.3%) | 87 | (14.5%) |  |
| Most Deprivation | 68 | (43.9%) | 185 | (30.9%) |  |
| **NDI Not Available** | 21 | (13.5%) | 55 | (9.2%) |  |
| * NDI ranges from -2.5 – 1.9; higher values indicate a greater level of neighborhood deprivation | | | | | |

Supplemental Table 1. Distance to Screening Site and Neighborhood Deprivation Index (NDI) Among Individuals in the Incomplete Screening and Complete Screening Subgroups

| **Supplemental Table 2. Baseline Characteristics Of Individuals Receiving Shared Decision Before and After Telehealth Implementation** | | | | | |
| --- | --- | --- | --- | --- | --- |
|  | **Pre-Telehealth Shared Decision Making**  **(n = 833)** | | **Post-Telehealth Shared Decision Making**  **(n = 754)** | | **p-value** |
| **Age, mean (SD)** | 64.28 | (5.82) | 63.93 | (5.73) | 0.223 |
| **Gender, n (%)** |  |  |  |  | 0.849 |
| Female | 470 | (56.4%) | 429 | (56.9%) |  |
| Male | 363 | (43.6%) | 325 | (43.1%) |  |
| **Ethnicity, n (%)** |  |  |  |  | 0.450 |
| Hispanic/Latinx | 36 | (4.3%) | 27 | (3.6%) |  |
| **Race, n (%)** |  |  |  |  | 0.163 |
| Black/African-American | 320 | (38.4%) | 307 | (40.7%) |  |
| White | 462 | (55.5%) | 416 | (55.2%) |  |
| Other^a^ | 51 | (6.1%) | 31 | (4.1%) |  |
| **Smoking status, n (%)** |  |  |  |  | 0.915 |
| Current | 464 | (55.7%) | 422 | (56.0%) |  |
| Former | 369 | (44.3%) | 332 | (44.0%) |  |
| **Pack-years, mean (SD)** | 54.42 | (25.61) | 51.96 | (23.50) | 0.046 |
| **Personal history of cancer, n (%)** | 163 | (19.6%) | 133 | (17.6%) | 0.343 |
| **Family history of lung cancer, n (%)** | 247 | (29.7%) | 219 | (29.0%) | 0.915 |
| **COPD, n (%)** | 372 | (44.7%) | 408 | (54.1%) | < 0.001 |
| **BMI, mean (SD)** | 29.10 | (6.95) | 28.61 | (7.70) | 0.183 |
| **Education, n (%)** |  |  |  |  | < 0.001 |
| <HS Diploma | 134 | (16.1%) | 80 | (10.6%) |  |
| HS Diploma/GED | 347 | (41.7%) | 350 | (46.4%) |  |
| >HS Diploma | 336 | (40.3%) | 287 | (38.1%) |  |
| Unknown | 16 | (1.9%) | 37 | (4.9%) |  |
| **Insurance Status, n (%)** |  |  |  |  | 0.920 |
| Medicare | 300 | (36.0%) | 278 | (36.9%) |  |
| Medicaid/Dual Eligible | 215 | (25.8%) | 189 | (25.1%) |  |
| Private/Other^b^ | 318 | (38.2%) | 287 | (38.1%) |  |
| **PLCOm2012 Risk, mean (SD)** | 6.66 | (6.05) | 6.34 | (5.64) | 0.320 |
| **Patient Type, n (%)** |  |  |  |  | < 0.001 |
| New Patient | 611 | (73.3%) | 360 | (47.7%) |  |
| Return Patient | 222 | (26.7%) | 394 | (52.3%) |  |
| **Neighborhood Deprivation Index, n (%)** |  |  |  |  | 0.030 |
| Least Deprivation | 128 | (15.4%) | 95 | (12.6%) |  |
| Below Average  Deprivation | 98 | (11.8%) | 89 | (11.8%) |  |
| Average Deprivation | 178 | (21.4%) | 138 | (18.3%) |  |
| Above Average  Deprivation | 130 | (15.6%) | 103 | (13.7%) |  |
| Most Deprivation | 218 | (26.2%) | 253 | (33.6%) |  |
| Neighborhood  Deprivation Index Not  Available | 81 | (9.7%) | 76 | (10.1%) |  |
| ^a^ Other race includes individuals who reported their race as Asian, Alaskan Native/American Indian, Native Hawaiian/Pacific Islander, or More than One Race.  ^b^ Private/Other insurance includes Private Insurance, State Marketplace, and Workers Compensation Plans | | | | | |

Supplemental Table 2. Baseline Characteristics Of Individuals Receiving Shared Decision Before and After Telehealth Implementation

**FIGURE LEGEND**

**Supplemental Table 1.** Distance to Screening Site and Neighborhood Deprivation Index (NDI) Among Individuals in the Incomplete Screening and Complete Screening Subgroups

**Supplemental Table 2.** Baseline Characteristics Of Individuals Receiving Shared Decision Before and After Telehealth Implementation
